# The Dilemma of Inadvertent Pontine Demyelinosis: A Review of Literature

**DOI:** 10.7759/cureus.3174

**Published:** 2018-08-21

**Authors:** Abu Baker Sheikh, Rao M Afzal, Shazib Sagheer, Marvi M Bukhari, Anam Javed, Adeel Nasrullah, Usman Tariq, Fahad Athar, Muhammad Sabih Saleem

**Affiliations:** 1 Internal Medicine, University of New Mexico, Albuquerque, USA; 2 Internal Medicine, Shifa College of Medicine, Islamabad, PAK; 3 Internal Medicine, University of New Mexico Hospital, Albuquerque, USA; 4 Internal Medicine, College of Medicine and Dentistry, University of Lahore, Lahore, PAK; 5 Internal Medicine, Allegheny General Hospital, Pittsburgh, USA; 6 Research Assistant, Yale University School of Medicine, New Haven, USA; 7 Other, Albert Einstein Medical Center , Philadelphia, USA; 8 Medicine, Shifa International Hospital, Islamabad, PAK

**Keywords:** osmotic demyelination syndrome, sodium correction

## Abstract

Osmotic demyelination syndrome is classically associated with a swift adjustment of previously low serum sodium levels which lead to cellular dehydration and subsequent neurological insult. We also review the epidemiology, different postulations to explain the underlying pathophysiology, current diagnostic modalities, subsequent therapeutic interventions used to manage this phenomenon, and the resultant prognosis of this ailment.

## Introduction and background

Osmotic demyelination syndrome (ODS) characteristically affects the central pons symmetrically, but can also affect other areas of the brain such as the basal ganglia, thalamus, and lateral geniculate bodies. It is mainly observed in patients with underlying issues such as alcoholism, malnourishment, and/or a previously unceasing illness. However, the most common affiliate is robust fluid and electrolyte replenishment as most patients have hyponatremia as a frequent affiliate [1]. 

## Review

Epidemiology

Osmotic demyelination syndrome (ODS) is a noninflammatory demyelinating disease that usually involves the base of the pons (central pontine myelinolysis, CPM) and may often extend to the extrapontine structures (extrapontine myelinolysis, EPM) [1-4]. It is a rare condition that has an undetermined incidence, with many cases diagnosed via autopsy [5]. Although it was first described by Adams et al. as a solely pontine entity, an autopsy study in 1987 that included 58 cases of ODS described the presence of CPM in half, a combination of CPM and EPM in 30%, and isolated EPM in 20% of the cases [6-7]. However, in clinical practice, EPM is a rare find with an incidence of less than 10% in patients suffering from CPM. An important factor contributing to this rarity is the masking of EPM symptoms by a concomitant pontine dysfunction [8-9].

Clinical presentation

The clinical presentation of ODS is highly variable and dependent on the region of the brain that is affected. The spectrum of presentations ranges from an asymptomatic clinical picture to severe outcomes such as coma and death. The typical presentation is of paraparesis or quadriparesis accompanied by symptoms of pseudobulbar palsy including dysarthria and dysphagia [5]. CPM can also lead to ‘locked-in syndrome,’ a state of preserved consciousness along with the paralysis of all voluntary movement except the eyes. On the other hand, EPM can present with cognitive dysfunction, myoclonus, dystonia, parkinsonism, and choreoathetosis [8, 10-11]. In some cases, the only evidence of EPM is nonspecific symptoms such as lethargy or altered mood which underscores the need for a high clinical suspicion in the setting of vague symptoms in patients undergoing an electrolyte replenishment [12].

Pathophysiology

The precise pathophysiology of this syndrome is yet to be fully elucidated. Postulated theories include the adaptation of the brain to chronically low levels of the intracellular osmolyte. A subsequent exposure to hypertonic stress resulting from a rapid correction of hyponatremia causes the ions to quickly re-enter the intracellular space and compels the water to follow. As this occurs, the intracellular sodium and chloride levels rise to a higher than normal value resulting in cellular dehydration [1, 13-15]. Disruption of the blood-brain barrier is also considered to be a key component in the pathogenesis, followed by oligodendrocyte degeneration, an influx of macrophages, and degradation of myelin [14-16]. ODS occurs most frequently due to osmotic damage following a rapid correction of hyponatremia which is defined as a correction by more than 12 mEq/L/day and/or 18 mEq/L in 48 hours [17]. However, it is not solely due to rapid correction of hyponatremia, as there have been reported cases, albeit rare, such as ours, in which ODS developed despite correction of serum sodium according to the proposed guidelines [18].

Diagnosis

The main diagnostic modality employed is a brain magnetic resonance imaging (MRI) scan. T1-weighted images will show the symmetric hypointense lesions, meanwhile T2-weighted images have symmetric hyperintense lesions [19-21]. The characteristic “bat-winged” or “trident-shaped” appearance in the center of the pons is the classic finding on MRI for CPM [22]. Moreover, a retrospective study concluded that patients with high clinical suspicion of ODS can aid from serial MRIs as initial MRIs findings may be unrevealing [23].

Treatment and prognosis

Despite the proposal of numerous treatment methods via case series and case reports including thyrotropin-releasing hormone, plasmapheresis, steroids, and immunoglobulins; there is a glaring paucity of data due to the absence of any large-scale studies regarding effective treatment guidelines for ODS. Hence, the mainstay of treatment of ODS is supportive along with preventing the development of secondary complications [5, 24-27]. The outcome of ODS has considerable variations that include an almost complete recovery to a nominal improvement of the resultant clinical manifestations [28-29]. Most of the early studies maintained that ODS has an inevitably grave prognosis [6, 7, 30]. However, more recent studies have demonstrated strikingly distinct results [23, 31-32]. McCormick et al. indicated almost 100% mortality within three months after admission following ODS in 1967, whereas in 2011, Graff-Radford et al. showed a favorable outcome in 60% of the cases with a mortality of 8% in the acute setting [23, 33]. We postulate that rapid detection of ODS with MRI techniques and a subsequently prompt initiation of treatment, coupled with new advances in management modalities could explain the improvement in overall mortality.

Normal sodium correction and ODS

We conducted a review of the literature using PubMed to ascertain the reported cases of ODS in patients with a normal sodium correction from 2003 to 2018. We identified a total of six cases previously reported which are summarized in Table 1.

**Table 1:** Review of the literature using PubMed summarizing the reported cases of osmotic demyelination syndrome in patients with a normal sodium correction from 2003 to 2018. M, male; F, female; MRI, magnetic resonance imaging; CPM, central pontine myelinolysis; ODS, osmotic demyelination syndrome.

Publication	Age	Sex	Clinical presentation	Sodium concentration at time of presentation (mEq/L)	Rate of sodium correction (mEq/L/day)	Onset of ODS presentation	Diagnostic modality	Treatment	Outcome
Orakzai et al. [34]	52	M	Jaundice and confusion, history of alcohol abuse since 35 years	122	<12	Deterioration of mental status, conscious but only responding to painful stimuli, sluggish pupillary responses, bilateral upgoing plantars	After an initial unremarkable MRI, a repeat MRI four weeks later showed abnormal T2 prolongation of the central pons with a lack of enhancement	Neuro-rehabilitation with supportive care and physical therapy	Marked improvement in the mental status and the ability to move all four limbs
Koul et al. [35]	47	M	Altered sensorium	94	8	Deterioration of consciousness and quadriparesis leading to ‘locked-in’ syndrome	MRI revealed symmetrical hypointense areas on T1 and hyperintense areas on T2 in the pons and basal ganglia	Supportive	Unknown
Hu et al. [36]	30	M	Upper gastrointestinal hemorrhage	114.8	<5	Paresis of the upper limbs, dysphagia, and dysarthria	MRI showing bilateral basal ganglia lesions	Slower correction of hyponatremia, thiamine, cobalamin, folate, and multivitamin supplements	Recovered
Pietrini et al. [37]	61	F	Stomach ache, nausea, vomiting, and drowsiness	103	12 in the first day, reduced to 3-4 for the ensuing days of treatment	Moderate quadriparesis, bilateral tremors, and limb dysmetria	MRI showing symmetrical areas of signal hyperintensity on T2 in the central pons	High dose steroid therapy followed by intravenous immunoglobulins	Died 18 days after the onset of CPM due to a massive pulmonary embolism that was discovered during an autopsy
Dellabarca et al. [38]	69	M	Weakness, falling and confusion for the previous one week; history of alcoholism	109	<10.5	Deterioration of mental status, dysarthria, difficulty in swallowing, bilateral cogwheel rigidity, and dysdiadochokinesia	MRI showing symmetrical central pontine lesions along with symmetrical lesions in the basal ganglia	Palliative care	Death due to respiratory failure secondary to a suspected aspiration pneumonia
Georgy et al. [39]	37	F	Loss of consciousness	105	<8	Widespread muscle weakness, dysarthria, and difficulty in swallowing	MRI showed T2-weighted hyperintensity in the pons	Supportive care with physical rehabilitation	Considerable improvement as patient started walking with minimal assistance and recovered her ability to swallow. There was a retention of mild dysarthria

The most common presentation was that of altered mentation and loss of consciousness. Deterioration was depicted by later developments such as dysphagia, dysarthria, and paresis of the limbs. Most of the patients were managed with supportive measures. The clinical outcome was favorable in half the patients who eventually recovered while the remaining patients succumbed to the initial neurological insult.

## Conclusions

Osmotic demyelination syndrome is a rare clinical entity. It has an undetermined clinical incidence with most patients diagnosed during an autopsy. The initial neurological insult occurs secondary to a rapid replenishment of sodium; however, a normal correction could also precipitate the syndrome in rare cases. Nonspecific to detrimental symptoms underscore the ambiguous nature of the clinical presentation. The MRI scans aid in the diagnosis which should be followed by prompt treatment. However, despite recent advances in clinical management, the outcomes remain obscure.
